# Microstructure and Properties of Al*_x_*Cr_1−*x*_CoFeNi High-Entropy Alloys Prepared by Spark Plasma Sintering

**DOI:** 10.3390/ma18040755

**Published:** 2025-02-08

**Authors:** Gang Li, Xiangran Meng, Chunpin Geng, Chongshuo Wang, Haifang Ren, Xiaoying Guo, Sinan Li, Ying Tao

**Affiliations:** 1 College of Mines, Liaoning Technical University, Fuxin 123000, China; stars2387@vip.sina.com; 2 School of Materials Science and Engineering, Yingkou Institute of Technology, Yingkou 115000, China; 15114226954@139.com (C.G.); 13065357759@163.com (C.W.); renhaifang1988@163.com (H.R.); 3 School of Metallurgy and Materials Engineering, Liaoning Institute of Science and Technology, Benxi 117004, China; wgzxhy@126.com (X.G.); lisinan@lnist.edu.cn (S.L.); taoying@lnist.edu.cn (Y.T.)

**Keywords:** high-entropy alloys, mechanical alloying, spark plasma sintering, tensile properties, friction wear performance, Tafel testing

## Abstract

CoCrFeNi high-entropy alloys represent a novel structural material with considerable application potential in a variety of fields, including aerospace, automobiles, ships, machining, energy, soft magnetic materials, and hydrogen storage materials. The present study investigates the impact of the Al element on the structure and properties of the alloy. The preparation of the Al*_x_*Cr_1−*x*_CoFeNi (*x* = 0.1, 0.2, 0.3, 0.4, 0.5) powders involved the use of a variety of elemental metal powders as raw materials and a mechanical alloying process at 350 rpm for 40 h. The sintering of the alloy powders was subsequently conducted using spark plasma sintering at 1000 °C. The microstructure of the alloys was analyzed using XRD, SEM, and EDS, and the properties were analyzed using a universal testing machine, a hardness measurement, friction and wear measurement, and an electrochemical workstation. The study shows that when *x* = 0.1, the crystal structure of Al_0.1_Cr_0.9_CoFeNi consists of a double FCC phase and a trace amount of σ phase. As the aluminum content increases, part of the FCC phase begins to transform to BCC. When *x* = 0.2~0.5, the alloy consists of a double FCC phase and a BCC phase and a trace amount of a sigma phase. As the BCC phase in the alloy increases, the tensile strength of the alloy increases, the ability to deform plastically decreases, and the hardness increases. The highest ultimate tensile strength of 1163.14 MPa is exhibited by Al_0.5_Cr_0.5_CoFeNi, while the minimum elongation is 26.98% and the maximum hardness value is 412.6 HV. In the initial stage of friction measurement, the wear mechanism of Al*_x_*Cr_1−*x*_CoFeNi is adhesive wear. However, as the test time progresses, an oxide layer begins to form on the alloy’s surface, leading to a gradual increase in the friction coefficient. At this stage, the wear mechanism becomes a combination of both adhesive and abrasive wear. Once the oxidation process and the wear process have reached a dynamic equilibrium, the friction coefficient stabilizes, and the wear mechanism transitions to a state of abrasive wear. The Al_0.1_Cr_0.9_CoFeNi alloy demonstrates the lowest friction coefficient and wear rate, exhibiting values of 0.513 and 0.020 × 10^−3^ mm^3^/Nm, respectively, while the Al_0.5_Cr_0.5_CoFeNi alloy demonstrates the highest performance, with a self-corrosion voltage of 0.202 V in a 3.5 wt.% NaCl solution. The experimental findings demonstrate that, in the presence of a decline in the Cr element within a high-entropy alloy, an augmentation in the Al element can facilitate the transition of the FCC phase to the BCC phase within the alloy, thereby enhancing its mechanical properties. However, in the Al*_x_*Cr_1−*x*_CoFeNi HEAs, the presence of the Cr-rich and Cr-poor phases invariably results in selective corrosion in a neutral NaCl solution. The corrosion resistance of this alloy is weaker than that of a single-phase solid solution alloy with a similar chemical composition that only undergoes pitting corrosion.

## 1. Introduction

High-entropy alloys (HEAs), a novel class of multi-principal element alloys, brought a new era in the study of alloy materials [1]. The presence of five or more elements in close proximity to the iso-atomic ratio endows alloys with high configurational entropy, which confers upon HEAs the capacity to form stable simple solid solution phases. This, in turn, endows HEAs with exceptional mechanical properties and commendable corrosion resistance [2]. This property gives high-entropy alloys excellent mechanical and physical properties, including high strength, high fracture toughness, and the ability to conduct electricity at very low temperatures [3]. In addition, they exhibit excellent resistance to high-temperature oxidation and corrosion. Consequently, in the aerospace field [4], where the temperature of service varies widely, in the field of ultra-high temperature structural materials [5], in marine industrial equipment [6], and in the nuclear industry [7], where there are harsh working conditions, high-entropy alloys have a wider range of application prospects than traditional alloys due to their rich elemental composition and flexible selection of elemental content.

Among the numerous HEA systems, the single-phase face-centered cubic (FCC) structure of CoCrFeNi-based high-entropy alloys exhibits remarkable plasticity, machinability and corrosion resistance, as evidenced by research [8]. Structural materials for underground mining machinery, such as crankshafts, gears, and conveyor belt linings, require materials that have high hardness, strength, good wear resistance, and corrosion resistance at room temperature [9]. CoCrFeNi-based high-entropy alloys also have great potential for use in mining structural materials and wear-resistant mining coatings. FCC equiatomic FeCoNi alloys with a grain size of approximately 10 nm and a thickness of approximately 0.2 mm were prepared by electrodeposition in an aqueous solution, exhibiting ultimate tensile strengths up to 1.6 GPa [10]. It is anticipated that this material will emerge as a novel structural option for use in environments prone to corrosion. Although the strength and hardness of the alloy are relatively low, these properties can be significantly enhanced by the addition of elements [11,12], particle dispersion strengthening [13], optimizing the preparation process [10,14], and heat treatment, among other methods, provided that the plasticity of the alloy is maintained. This will facilitate the wider application of the FCC phase HEAs in industrial production and life. In the field of research into high-entropy alloys, the common modification methods are the increase in the types of elements and the adjustment of the element content. It has been established that for Al*_x_*CoFeNi alloys prepared using the vacuum melting process, as the Al content *x* changes from 0.25 to 1, the microstructure of the alloys changes from FCC to BCC, the yield strength increases from 158.4 MPa to 967.4 MPa, and the toughness decreases [15]. Furthermore, it has been demonstrated that the Cr element can significantly enhance the strength of Co_40_Fe_20_Ni_40−*x*_Cr*_x_* HEAs obtained by the vacuum melting process, with the yield strength of Co_40_Fe_20_Ni_10_Cr_30_ being approximately 50% higher than that of Co_40_Fe_20_Ni_30_Cr_10_ [16]. Furthermore, an increase in Al content during the vacuum-melting process results in a transition from an FCC+BCC phase to a B2 phase, characterized by distinct chemical compositions. This transition is accompanied by an enhancement in the hardness of the alloy, with Al_1.25_Cr_0.75_CoFeNi demonstrating the highest Vickers hardness value of 515 HV [17].

The microstructure and properties of HEAs are directly related to the preparation process. Mechanical alloying (MA) is a process of producing homogenized nanoalloy powders. It is a widely utilized method for the preparation of nanocrystalline high-entropy alloy powders. The process of mechanical alloying has been demonstrated to enhance both solubility and configurational entropy [11]. During mechanical alloying, factors such as rotational speed, milling time, ball size, and ball-to-material ratio [18] directly impact the alloying effect. Spark plasma sintering (SPS) is an advanced powder metallurgy method for solidifying high-entropy alloy powders. The high-energy plasma during the sintering process enables rapid heating of the alloy powder, resulting in a fine and uniform microstructure [19]. Furthermore, the alloy remains in the high-temperature region for a brief period, which is conducive to the maintenance of the substable structure.

As-cast CoCrFeNi high-entropy alloys typically exhibit an FCC phase structure. The higher melting point and larger atomic radius of the Cr element impart a proclivity for the CoCrFeNi-based alloys to form a BCC phase. The non-equilibrium solidification process, mechanical alloying, and other techniques facilitate the formation of a substable BCC phase, which tends to increase with the increase in the content of the Cr element. When the Cr content in an alloy is elevated in certain regions, a high Cr σ phase is formed at the grain boundaries. The Al element has a stronger bonding affinity with CoCrFeNi high-entropy alloys with larger atomic radii, and its incorporation into the alloying system intensifies the lattice distortions. This enables the fabrication of HEAs with exceptional mechanical properties, including FCC+BCC or BCC+B2 dual phases, as evidenced by references [17,20,21]. In conclusion, the increase in the content of Al and Cr elements in the HEA system will result in an increased tendency for the FCC to BCC transformation. A significant number of studies have examined the influence of Al content on the microstructure and properties of CoCrFeNi-based HEAs. These studies have demonstrated that the introduction of Al into the alloys without altering the original alloying system may superimpose the effects of Al and Cr elements, thereby promoting the phase transition. In order to mitigate this effect, the present work employs the replacement of a portion of the Cr elements in the alloy system with Al elements, and the preparation of Al*_x_*Cr_1−*x*_CoFeNi (*x* = 0.1, 0.2, 0.3, 0.4, 0.5) high-entropy alloys by the MA+SPS technique.

In order to expand the application of CoCrFeNi high-entropy alloys in underground mining equipment, an alloy with both ductility and strength as well as hardness is designed. In this study, Al was introduced into the CoCrFeNi alloy in the form of a small amount of Al replacing Cr in an equivalent molar amount to investigate the effect of Al on the microstructure and properties of the CoCrFeNi alloy. This provides insight into the design and performance control of Al*_x_*Cr_1−*x*_CoFeNi HEAs and supports their application in underground mining scenarios.

## 2. Materials and Methods

### 2.1. Material Preparation

The weight percentages of the required Al, Cr, Co, Fe, and Ni metal powders were calculated based on the chemical composition of the Al*_x_*Cr_1−*x*_CoFeNi HEAs (as shown in Table 1). The metal powders used in the experiment had a purity greater than 99.9% and a particle size less than 25 µm (Zhongmai Metal Materials Co., Ltd., Zhongshan, China). The weight of each sample was 20 g. As demonstrated in Table 2, the valence electron concentration (*VEC*), atomic size, and mixing enthalpy Δ*H*_mix_ of the elements are presented. The atomic size of Co, Fe, and Ni are comparable, thereby minimizing the lattice distortion energy and facilitating the formation of a single solid solution phase. The Δ*H*_mix_ of Co-Fe, Co-Ni, and Fe-Ni approaches or equals 0 KJ/mol, a factor that contributes to the formation of an ordered solid solution. Research has demonstrated that the entropy alloy with an equal molar ratio of CoFeNi is an FCC phase solid solution alloy [8,10]. The low atomic size difference and mixing enthalpy characteristics of the ternary CoFeNi provide a stable solid solution base for Al*_x_*Cr_1−*x*_CoFeNi.

According to the parameters related to high-entropy alloys calculated in Table 1, the addition of Al to the CoCrFeNi system caused an increase in the mixing entropy (∆*G*_mix_) of the alloys from 1.47 R to 1.56 R (R is the gas constant, 8.314 J/(K·mol)), a decrease in the mixing enthalpy (∆*H*_mix_) from −4.98 to −9.38 KJ/mol, and an increase in the difference in atomic radii (*δ*) from 2.30% to 4.47% [22]. Combined with the phase prediction criterion for high-entropy alloys, the crystal structures of the Al*_x_*Cr_1−*x*_CoFeNi HEAs were transformed from the FCC phase to the FCC+BCC phase.

In the CoCrFeNi HEAs, which is typically the FCC phase, substituting the Cr element with Al, which possesses a larger atomic radius, a more negative mixing enthalpy, and a smaller *VEC*, can enable the customization of the alloy structure and properties by modulating the ratio of Al and Cr elements. According to the calculated parameters of the high-entropy alloy in Table 3, when *x* increases from 0.1 to 0.5, the mixing entropy (∆*G*_mix_) of the Al*_x_*Cr_1−*x*_CoFeNi HEAs increases from 1.47 R to 1.56 R (R is the gas constant, 8.314 J/(K·mol)), the mixing enthalpy (∆*H*_mix_) decreases from −4.98 KJ/mol to −9.38 KJ/mol, the difference in atomic radii (*δ*) increased from 2.30% to 4.47%, and *VEC* decreased from 8.18 to 7.50 [22]. The formation of a stable disordered solid solution phase is contingent upon the following criteria: −7.27 KJ/mol < ∆*H*_mix_ < 4 KJ/mol and *δ* < 4.27%. Accordingly, when *VEC* > 8, the phase is identified as FCC; 6.8 < *VEC* < 8, the phase is designated as FCC+BCC; and *VEC* < 6.8, the phase is recognized as BCC. Combined with the phase prediction criterion for high-entropy alloys, the crystal structures of the Al*_x_*Cr_1−*x*_CoFeNi (*x* = 0.1 ~ 0.5) HEAs were transformed from the FCC phase to the FCC+BCC phase. When *x* = 0.5, *δ* > 4.27%, and the possibility of an ordered phase in the alloy phase increases, which has the potential to reduce the mechanical properties of the alloy. Therefore, the present study considers only five alloy compositions with *x* = 0.1 ~ 0.5.

### 2.2. Experimental Process

**Figure 1:**
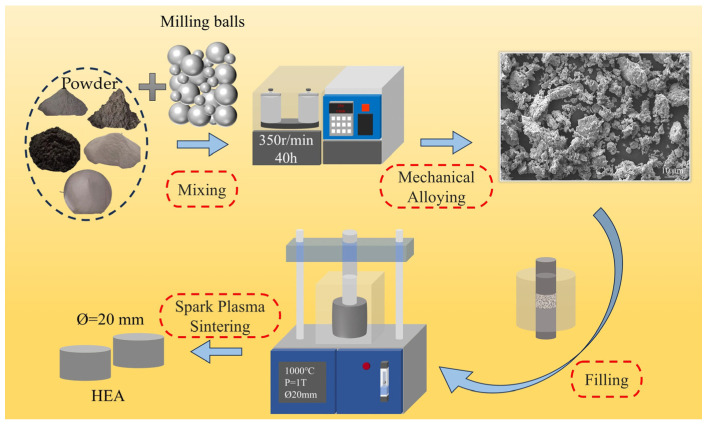
Experimental schematic for the preparation of HEAs.

The metal powders were subjected to mechanical alloying in a high-energy planetary ball mill (F-P4000E, Focucy, Changsha, China) for 40 h at 350 rpm. The grinding balls were 3 mm and 10 mm in diameter and comprised hard stainless steel. The mass ratio of the grinding balls to the metal powder was 1:1. The weight ratio of the metal powder to the grinding balls was 1:5. The ball-milled powders were subsequently subjected to sintering in a discharge plasma sintering furnace (SPS, 100T-20-III, Chenhua Electric Furnace Co., Ltd., Shanghai, China). The sintering temperature was 1000 °C, the holding time was 15 min, and the applied pressure was 31.2 MPa. The sintered specimen was a cylinder with a diameter (Ø) of 20 mm and a height (h) of 8 mm. Figure 1 depicts the experimental schematic for the preparation of HEAs.

The alloy specimens were subjected to corrosion using a diluted aqua regia solution (HCl:HNO_3_ = 1:3) for a period of 10 to 15 s following a pre-grinding and polishing treatment. The physical phase of the alloys was analyzed by X-ray diffraction (XRD) using a Cu target and a Bruker Advance D8 XRD analyzer (Bremen, Germany). The X-ray diffraction (XRD) tests were conducted at a tube current of 40 mA, a tube voltage of 40 kV, and a scanning angle of 10° to 90°. The microstructure of the specimens were characterized by scanning electron microscopy (SEM, ZEISS, EVO-18, Oberkochen, Germany) and energy spectrometry (EDS, Oxford Instruments, X-max, Oxford, UK). A transmission electron microscope (TEM, FEI Talos-F200s, Houston, TX, USA) was utilized to examine the microstructure and crystal structure of the high-entropy alloy powder.

The tensile properties of the HEAs at room temperature were tested using a universal materials testing machine (Instron, Model 68TM-50, Norwood, MA, USA), with a test temperature of 25 °C and a tensile speed of 2 mm/min. The hardness of the alloys was quantified by means of a Vickers hardness tester (Huayin Testing Instruments Co., HVS-50, Laizhou, China) with a load of 10 kgf and a holding time of 10 s. The friction properties of the alloys were analyzed at room temperature by means of a friction and wear tester (RTEC Instruments, MFT-5000, San Jose, CA, USA). The counter-wear materials utilized in the tests were GCr15 grinding balls with Ø 6 mm. The dimensions of the alloy specimen were Ø7 mm × 3 mm in thickness. The test was conducted at a rotational speed of 120 r/min for a duration of 600 s, with a load of 10 N and a friction radius of 1.5 mm.

The electrochemical properties of the alloys were investigated utilizing a three-electrode system in conjunction with an electrochemical workstation (Princeton Applied Research, PARSTAT 4000A, Oak Ridge, TN, USA). The experiments were conducted at 25 °C in a 3.5 wt.% NaCl solution, during which the alloy specimen, saturated calomel (SCE) and Pt served as the working, reference and auxiliary electrodes, respectively. The dynamic potential polarization curves (Tafel curves) of the alloys were tested after stabilization of the open-circuit potentials at test voltages of −400 mV to 400 mV and scanning speeds of 2 mV/s. Following the completion of the tests, the microstructure of the alloys was characterized by SEM and EDS analyses.

## 3. Results and Discussion

### 3.1. Phase and Microstructure of Al_x_Cr_1−x_CoFeNi

**Figure 2:**
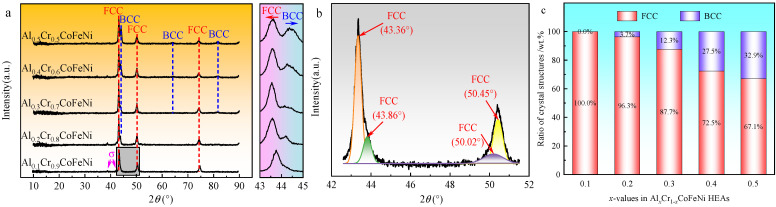
(**a**) XRD patterns of Al*_x_*Cr_1−*x*_CoFeNi HEAs. (**b**) Deconvolution of diffraction peaks in the region marked by a gray rectangular zone. (**c**) Ratio of crystal structures in Al*_x_*Cr_1−*x*_CoFeNi HEAs.

The XRD patterns of the Al*_x_*Cr_1−*x*_CoFeNi HEAs are shown in Figure 2a. The diffraction peak angles and peak intensities of the FCC phase identified in the figure were similar to the standard diffraction diagram (PDF#04-0850) for Ni, corresponding to crystallographic indices (1 1 1), (2 0 0), and (2 2 0). Similarly, the BCC phase corresponded to the standard diffraction diagram for Cr (PDF#06-0694) with crystallographic indices (1 1 0), (2 0 0), and (2 1 1). As shown in Figure 2a, the alloy consisted of FCC + σ phase when *x* = 0.1 and BCC peaks were observed in the XRD pattern when *x* ≥ 0.2. As the value of *x* increased, new diffraction peaks of BCC phase were observed in the XRD pattern and the peak intensity gradually increased. Following the implementation of peak fitting of the XRD pattern of the high-entropy alloy using Jade 6.5 software, the weight percentages of the FCC and BCC phases in the alloy were calculated, with the results presented in Figure 2c. Due to the absence of an accurate PDF card, the two FCC phases in the Al_0.1_Cr_0.9_CoFeNi were calculated in unison. The sigma phase in the XRD spectrum is less abundant and was not included in the calculation. As demonstrated in Figure 2c, as the Al content *x* increases from 0.1 to 0.5, the mass percentage of the BCC phase in the Al*_x_*Cr_1−*x*_CoFeNi HEAs increases from 0% to 32.9%.

The XRD patterns of the alloys were found to be compatible with the phase prediction results. From the magnified spectra of the 43°~45° region in Figure 2a, it was observed that the main peak of the FCC phase gradually shifted to the left with the increment of x from 0.1 to 0.5, and part of the FCC phase transformed into the BCC phase. As with steel, the Al and Cr elements in the CoCrFeNi high-entropy alloy are BCC phase-forming elements [23]. As demonstrated in Table 2, the atomic size of the Al element exceeds that of the remaining four transition metals, and the atomic-level induced stress is amplified in higher atomic packing efficiency (APE) structures. It is well established that the BCC structure exhibits a lower APE in comparison to the FCC structure; furthermore, an increase in Al content is associated with an escalation in atomic-level stress. When the induced stress reaches a threshold, the FCC structure will transform to the BCC structure [15,24]. The atomic size of the Cr element exceeds that of Co, Fe and Ni; thus, an increase in the Cr content can also promote a similar structural transformation.

**Figure 3:**
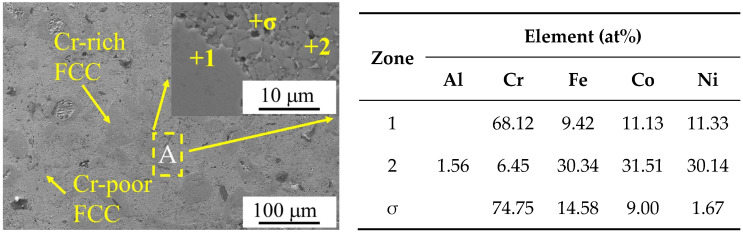
Microstructure and compositional analysis of Al_0.1_Cr_0.9_CoFeNi.

**Figure 4:**
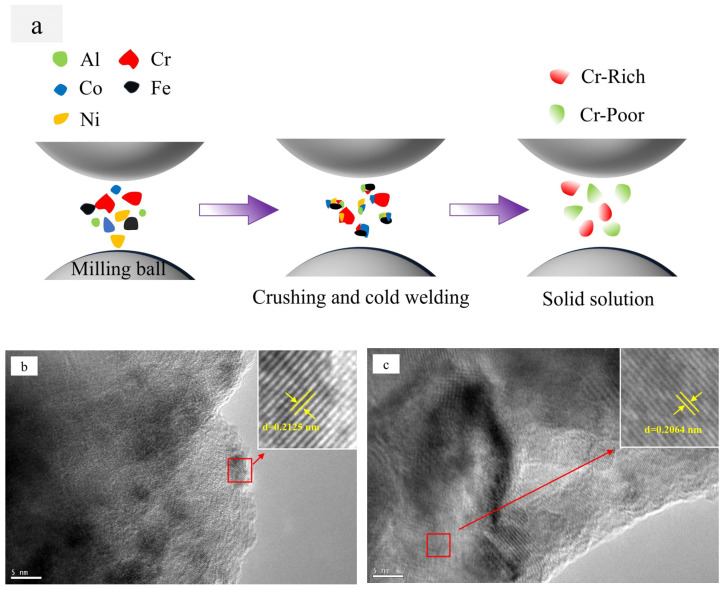
(**a**) Schematic diagram of the mechanical alloying process of Al_x_Cr_1−x_CoFeNi HEAs; HRTEM image of Al_0.1_Cr_0.9_CoFeNi powder. (**b**) FCC1 (d = 0.2125 nm). (**c**) FCC2 (d = 0.2064 nm).

Figure 2b shows the results of the back-convolution operation on the diffraction peaks in the gray region of Figure 2a [25]. Based on the results, it could be inferred that the XRD pattern of Al_0.1_Cr_0.9_CoFeNi was formed by the superposition of diffraction peaks with different structures. Combining the microstructure SEM images and the EDS results of the Al_0.1_Cr_0.9_CoFeNi alloy in Figure 3, the structure of the alloy consisted of two FCC phases with a large variation in Cr element content and tiny σ phases. In Figure 3, region 1 represents the Cr-rich FCC phase and region 2 represents the Cr-poor FCC phase. As illustrated in Figure 4, the mechanical alloying process involves subjecting metal powder to forces of friction and impact from milling balls, resulting in the fragmentation of the powder into fine particles. During the subsequent grain crushing and cold welding process, the Cr particles exhibit a significantly higher degree of hardness in comparison to the other four metal particles. The larger particles, which have not undergone complete crushing, cold weld with the smaller particles of the other metals. It is noteworthy that during this process, the Cr element exhibits signs of segregation. The diffusion of metallic elements can be categorized into two distinct processes: (1) Ni and Co dissolved into Fe of FCC to form the Cr-poor Fe (Ni, Co) FCC phase solid solution; and (2) Fe diffused into Cr to form the Fe-Cr BCC phase solid solution [26].

Figure 4b,c present high-resolution transmission electron microscope images of Al_0.1_Cr_0.9_CoFeNi high-entropy alloy powder, respectively. Figure 4b corresponds to the (1 1 1) crystal plane with a crystal plane spacing of d = 0.2125 nm in the FCC1 phase; Figure 4c corresponds to the (1 1 1) crystal plane with a crystal plane spacing of d = 0.2064 nm in the FCC2 phase. It is evident that there are two FCC phases with analogous crystal plane spacings in the Al_0.1_Cr_0.9_CoFeNi alloy, which is in accordance with the analysis results depicted in Figure 2b and Figure 3. The Al0.1Cr0.9CoFeNi alloy exhibits both Cr-rich and Cr-poor FCC phases.

Ding et al. [27] found that after replacing some of the Mn elements with Al elements in a CrMnFeCoNi high-entropy alloy with little interstitial concentration fluctuation, the atomic distribution of the alloy showed significant inhomogeneity, and obvious fluctuations in compositional concentration and frequent transverse slippage of dislocations were observed. Al and Cr elements contributed to the elemental bias phenomenon in Al*_x_*Cr_1−*x*_CoFeNi HEAs, and the metal atoms could only diffuse to a small extent during SPS sintering, which largely maintained the inhomogeneity of the elemental distribution. It can be hypothesized that this is the reason for the formation of the Cr-poor phase and the Cr-rich phase in the alloy structure.

**Figure 5:**
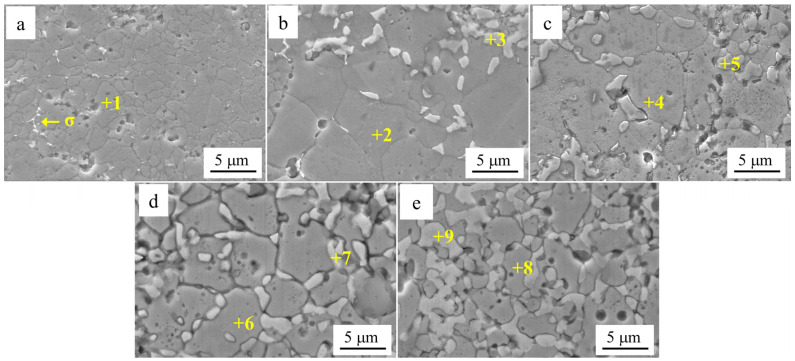
Microstructure of Cr-poor regions in Al*_x_*Cr_1−*x*_CoFeNi alloys: (**a**) *x* = 0.1, (**b**) *x* = 0.2, (**c**) *x* = 0.3, (**d**) *x* = 0.4, (**e**) *x* = 0.5.

Figure 5 shows the SEM images of Al*_x_*Cr_1−*x*_CoFeNi HEAs in the Cr-poor regions with *x* = 0.1, 0.2, 0.3, 0.4, 0.5 according to Figure 5a–e. Table 4 shows the EDS analysis results of the marked regions in Figure 5. As shown by the results of the SEM and EDS analyses, the Cr-poor region of the alloys consisted of isometric crystals of Cr-poor FCC and Cr-rich σ at *x* = 0.1. At *x* = 0.2, the Cr-poor region of the alloys consisted of isometric crystals of Cr-poor FCC, Cr-rich σ and Al-rich BCC. The number of BCC phases gradually increased as *x* increased from 0.1 to 0.5.

**Figure 6:**
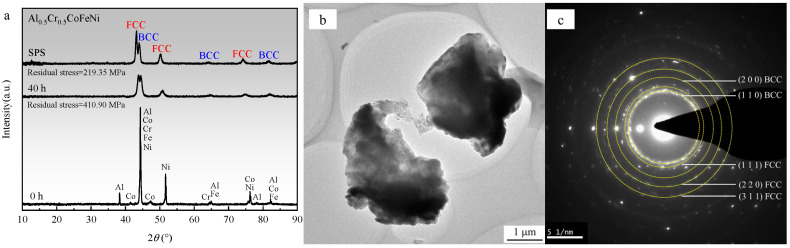
(**a**) XRD patterns of the as-mixed Al_0.5_Cr_0.5_CoFeNi powder, the alloy powder after 40 h of ball milling, and the bulk alloy after SPS sintering. (**b**) TEM bright-field image of the alloy powder after 40 h of ball milling. (**c**) SAED image of the alloy powder after 40 h of ball milling.

Figure 6a illustrates the XRD patterns of the Al_0.5_Cr_0.5_CoFeNi alloy in its as-received state, following 40 h of ball milling, and subsequent SPS sintering. It is evident from the figure that after 40 h of ball milling, the diffraction peaks of individual elements dissipate, giving rise to FCC and BCC solid solution diffraction peaks, thereby signifying the fundamental completion of the mechanical alloying process of the high-entropy alloy. The broadening of the diffraction peaks signifies a reduced grain size and elevated lattice distortion [28]. Following SPS sintering, the position of the diffraction peaks remains predominantly unaltered; however, the intensity of the FCC phase diffraction peaks increases, and the diffraction peaks become marginally narrower, indicating an augmented grain size. Utilizing the values of elastic Young’s modulus and Poisson’s ratio provided in references [29,30], the residual stress of the alloy powder post-ball milling was calculated to be 410.90 MPa employing Jade 6.5, and the residual stress of the bulk alloy post-SPS sintering was determined to be 219.35 MPa. It is evident that during mechanical alloying, significant levels of residual stress are generated within the powder particles due to collisions, fragmentation, and cold welding. The heat release and densification processes during SPS sintering serve to reduce the residual stress. Figure 6b,c present the TEM bright-field image and the selected area electron diffraction (SAED) pattern of Al_0.5_Cr_0.5_CoFeNi powder following 40 h of ball milling. As illustrated in Figure 6b, the particle size of the powder is approximately 3 mm. Figure 6c demonstrates that the SAED pattern reveals two distinct phases, which align closely with the XRD pattern analysis outcomes depicted in Figure 6a, representing the FCC and BCC phases, respectively.

### 3.2. Tensile Properties

**Figure 7:**
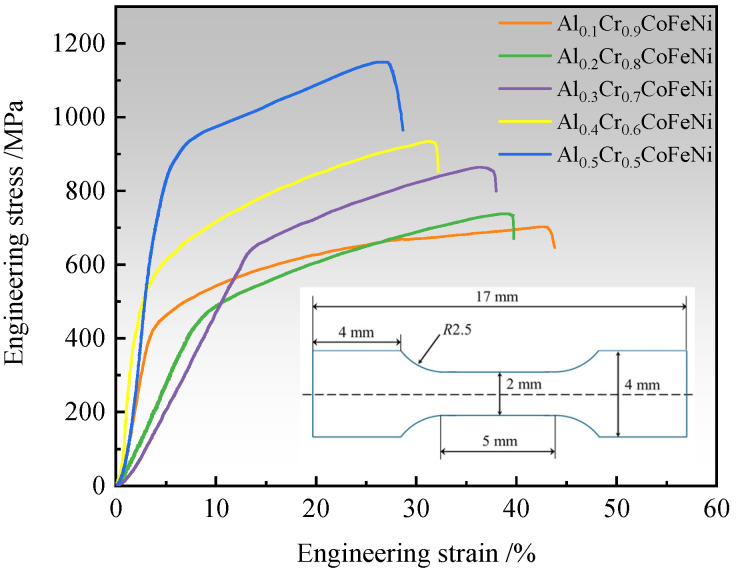
Engineering stress–strain curves at room temperature of Al*_x_*Cr_1−*x*_CoFeNi HEAs.

The mechanical properties of Al*_x_*Cr_1−*x*_CoFeNi HEAs were studied by means of room-temperature tensile testing. As illustrated in Figure 7, the engineering stress–strain curve of the alloy is presented. The yield strength (YS), ultimate tensile strength (UTS) and elongation obtained from the tensile curve are shown in Table 5. The outcomes of the tensile testing indicate a systematic enhancement in the UTS of the Al*_x_*Cr_1−*x*_CoFeNi HEAs with an increase in Al content, ranging from an initial value of 702.01 MPa at *x* = 0.1 to a maximum of 1163.14 MPa. Concurrently, the alloy’s elongation exhibited a concurrent decline, from 43.01% to 26.98%. The YS of the HEAs demonstrates a gradual increasing trend. However, the YS of Al_0.3_Cr_0.7_CoFeNi is higher than that of Al_0.4_Cr_0.6_CoFeNi, and only slightly lower than that of Al_0.5_Cr_0.5_CoFeNi. The solid solution structure of high-entropy alloys is directly related to their mechanical properties. The XRD spectrum analysis results indicate that as the Al content *x* increases from 0.1 to 0.5, the alloy’s phase transitions from FCC to FCC+BCC, with a concomitant gradual increase in the BCC phase content. The enhancement of the alloy’s tensile properties during plastic deformation is attributed to the interplay of two factors: the non-uniform distribution of composition and the interaction of two-phase grain boundaries [31]. The UTS of Al_0.5_Cr_0.5_CoFeNi is reported to be up to 1163.14 MPa. The mechanical properties of the Al*_x_*Cr_1−*x*_CoFeNi HEAs are comparable to the results of Xiong et al. [32], but lower than the compression properties of AlCoCrFeNi HEAs prepared using the MA+SPS process [33]. Two potential explanations for this discrepancy are proposed. Firstly, the manner in which the alloy is subjected to stress during the testing process differs. The SPS sintering process has been observed to yield alloys with residual stress and metastable phases [34], which have the capacity to influence the outcomes of testing. Secondly, the sintering temperature and pressure also affect the microstructure and properties of the alloy. These factors will be the primary focus of subsequent research in this paper.

### 3.3. Hardness and Wear Resistance

The Vickers hardness of Al*_x_*Cr_1−*x*_CoFeNi is listed in Table 6. Among the alloys, Al_0.5_Cr_0.5_CoFeNi exhibits the greatest Vickers hardness, reaching 412.6 HV, which is higher than that of FeCoCrNi prepared by a similar preparation process [35]. The number of BCC phases in Al*_x_*Cr_1−*x*_CoFeNi was found to be positively correlated with the aluminum content. The presence of fewer slip systems in the BCC phase than in the FCC phase results in less ease of dislocation movement. Consequently, Al_0.5_Cr_0.5_CoFeNi with a greater number of BCC phases exhibits higher hardness [15].

**Figure 8:**
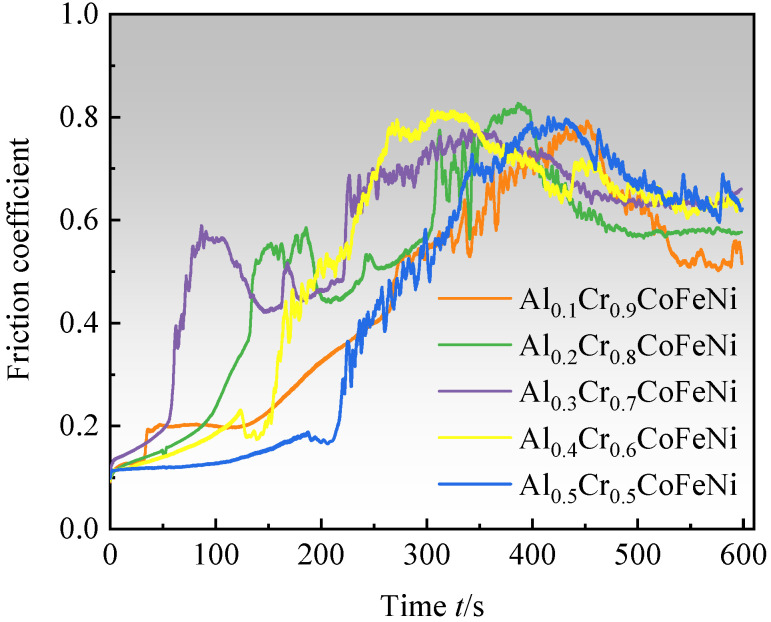
Friction coefficient–time curves of Al*_x_*Cr_1−*x*_CoFeNi alloys.

As illustrated in the graphical representation of the friction coefficient–time curves for the Al*_x_*Cr_1−*x*_CoFeNi HEAs (Figure 8), the experimental procedure is divided into three distinct phases. During the initial phase of the test (t ≤ 50 s), the predominant wear mechanism is adhesive wear. The friction coefficient begins at a low value and gradually increases as the grinding balls slide relative to the alloy surface, due to plastic deformation and solid welding, which causes the alloy particles to adhere to the surface of the grinding balls. (2) As the test progresses (50 s < t ≤ 400 s), the wear mechanism transitions to a combination of adhesive and abrasive wear. As a consequence of the generation of frictional heat, hard oxide particles begin to appear on the alloy surface, and these particles are also involved in the process of friction [36]. The surface of the alloy is severely worn, and the coefficient of friction is increasing and reaches its peak value. (3) In the final stage of the test (t > 400 s), oxidative wear becomes the main mechanism. The generation of heat due to friction leads to the formation of a sufficiently thick and continuous oxide layer consisting of various oxides on the alloy friction surface. This oxide layer serves to protect the alloy surface from adhesive and abrasive wear. At this point, the formation of the oxide film and the wear rate reach an equilibrium, and the coefficient of friction tends to stabilize after a slight decrease. When the oxide film is stabilized, the coefficient of friction tends to increase gradually with the increase in x-value, and the lowest coefficient of friction of 0.513 was found for Al_0.1_Cr_0.9_CoFeNi. The wear resistance of Al*_x_*Cr_1−*x*_CoFeNi is better than that of FeCoCrNi alloys produced by laser melting and cladding [37].

**Figure 9:**
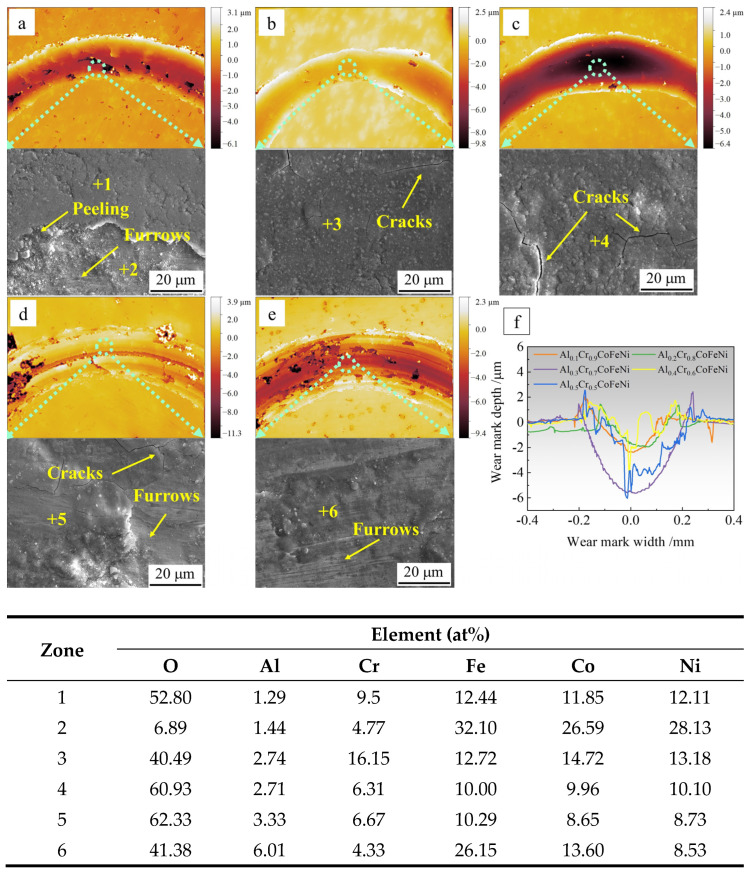
Morphology and compositional analysis of Al*_x_*Cr_1−*x*_CoFeNi HEAs wear marks: (**a**) *x* = 0.1, (**b**) *x* = 0.2, (**c**) *x* = 0.3, (**d**) *x* = 0.4, (**e**) *x* = 0.5, (**f**) longitudinal cross-section of abrasion marks.

Figure 9 presents the topographic images and compositional analysis of the wear marks of the Al*_x_*Cr_1−*x*_CoFeNi HEAs following the completion of the friction test. The values of *x* correspond to the following compositions: 0.1, 0.2, 0.3, 0.4, 0.5. In Figure 9a–e, respectively, the scale bar represents depth, with 0 mm corresponding to the position of the original plane of the alloy specimen. The area with discontinuous and darker colors in the wear marks indicates the position of the oxide film flaking off. The abrasion marks of oxide wear are relatively flat in Figure 9a–c, while the abrasion marks in Figure 9d,e contain grooves of varying depths. Figure 9f depicts a longitudinal cross-section of the abrasive wear marks. The wear rate of the alloy is calculated using the following formula: *K* = *V*/(*F* × *L*), where *V* is the wear volume, *F* is the normal force (set at 10 N), and *L* is the total sliding length [38]. The calculation results are presented in Table 7. As demonstrated in Figure 9f and Table 7, the abrasive grains of Al_0.1_Cr_0.9_CoFeNi and Al_0.2_Cr_0.8_CoFeNi exhibit superficial wear marks and possess a comparatively diminutive wear rate of approximately 0.020 × 10^−3^ mm^3^/Nm. The wear marks of Al_0.3_Cr_0.7_CoFeNi are the widest and deepest, and the wear rate is the largest, at 0.141 × mm^3^/Nm. In contrast, the depth of wear for Al_0.4_Cr_0.6_CoFeNi and Al_0.5_Cr_0.5_CoFeNi exhibits significant fluctuations, with a higher wear rate compared to Al_0.1_Cr_0.9_CoFeNi and Al_0.2_Cr_0.8_CoFeNi. The EDS analysis of the midpoint of the abrasive marks revealed a high concentration of oxygen (O) elements, indicative of an oxide film resulting from oxidative wear. This finding was corroborated by the analysis of the friction coefficient–time curves.

The SEM images of the friction positions and the EDS analysis results of the marked points demonstrate that the friction positions contain a considerable number of O elements. The research results cited in references [36,37,38] demonstrate that during oxidative friction, an oxide layer composed of various metal oxides, including Al, Fe, and Cr, forms on the surface of the abrasion marks of the AlCoCrFeNi HEAs. Furthermore, the even fine abrasion marks on the oxide film can be clearly observed in Figure 9d,e, which are the typical characteristics of oxidative wear and coincide with the results of the analysis of the friction coefficient–time curves. In Figure 9a, position 1 is the oxide film, and position 2 is the surface of the alloy matrix, and fine abrasion marks can be observed. The oxide film generated during the friction process is harder and more brittle than the alloy, and it is susceptible to rupture and the formation of cracks, or even spallation, when the temperature is repeatedly raised and lowered during the friction process [39]. The test results indicate that the Al_0.1_Cr_0.9_CoFeNi alloy exhibits the lowest friction coefficient and wear rate, demonstrating superior wear resistance. As indicated by the research findings of Vo and Deng [36,38], during the initial stage of alloy oxidation at the friction trace, the elements Al and Cr oxidize, resulting in the formation of an Al_2_O_3_ or Cr_2_O_3_ oxide layer. At this stage, the uniformity and continuity of the oxide layer phase have the greatest impact on the friction process. During the initial stage of oxidation friction, an oxide layer with Cr_2_O_3_ as the main component will first form on the surface of the Al_0.1_Cr_0.9_CoFeNi alloy. This connected and dense oxide layer protects the alloy substrate during friction. Subsequent friction processes involve the oxidation of additional metal elements, leading to the formation of an oxide layer comprising a mixture of metal oxides. The oxide layer’s composition undergoes changes during the process, with the Al element increasing the prevalence of Al_2_O_3_ and Cr_2_O_3_ in the initial layer. During friction, the different phases experience varying levels of strain, resulting in the fragmentation of the oxide layer. These fragments act as abrasive particles, leading to enhanced wear on the substrate.

### 3.4. Electrochemical Performance

**Figure 10:**
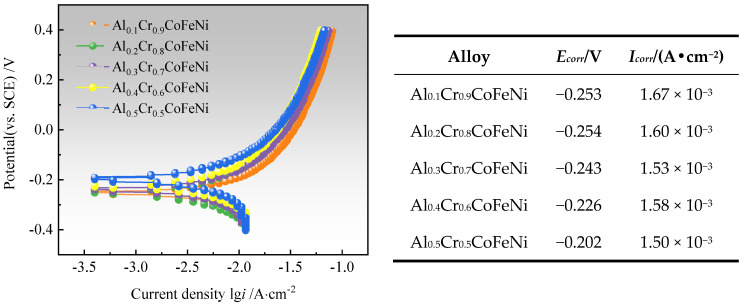
Tafel test curves of Al*_x_*Cr_1−*x*_CoFeNi HEAs.

Figure 10 illustrates the Tafel test curves of Al*_x_*Cr_1−*x*_CoFeNi HEAs in a 3.5 wt.% NaCl solution, along with the self-corrosion potential (*E*_corr_) and the self-corrosion current density (*I*_corr_) of the alloy, which were calculated by the extrapolation method [40]. It was observed that the value of *E*_corr_ was maximum at −0.202 V when *x* = 0.5, and Al_0.5_Cr_0.5_CoFeNi exhibited the least tendency to corrode in neutral solution [41]. Its corrosion resistance was slightly superior to that of Al_0.5_CuFeNiCoCr alloy prepared by powder metallurgy by Jiang et al. [42].

Al*_x_*Cr_1−*x*_CoFeNi HEAs invariably contain both a Cr-rich phase and a Cr-poor phase. In a 3.5 wt% NaCl solution, the Cr-rich phase functions as the cathode, while the Cr-poor phase functions as the anode. Due to the uneven distribution of the Cr element, a substantial potential difference exists between the two phases, thereby forming a micro-electrical couple. The dissolution of the anode phase is preferential, leading to a substantial surge in the self-corrosion current density. Consequently, the electrochemical corrosion process manifests as selective corrosion. The single-phase high-entropy alloy exhibits a uniform composition and structure, is devoid of local potential differences, exhibits a weak galvanic effect, and has a relatively low self-corrosion current. The electrochemical corrosion process is predominantly pitting corrosion [43]. Based on Faraday’s law, it was known that the density of corrosion current and the corrosion rate is proportional to the relationship. The corrosion rate of Al_0.5_Cr_0.5_CoFeNi was minimum, and its corrosion resistance was better than other alloys in this work.

**Figure 11:**
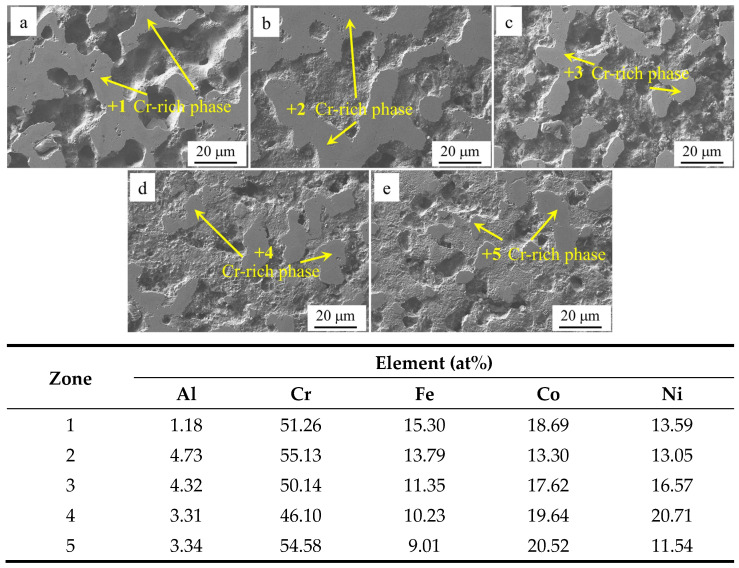
Microstructure of Al*_x_*Cr_1−*x*_CoFeNi alloys after Tafel testing: (**a**) *x* = 0.1, (**b**) *x* = 0.2, (**c**) *x* = 0.3, (**d**) *x* = 0.4, (**e**) *x* = 0.5.

Figure 11 presents the SEM images of the surface of Al*_x_*Cr_1−*x*_CoFeNi HEAs and the results of the EDS analysis of the marked areas following the Tafel test. The comprehensive analysis indicates that the potential of the Cr element in neutral solution NaCl is higher than that of the Al and Fe elements. The Cr-rich FCC phase is the cathode, and the cathodic reaction is 1/2O_2_ + H_2_O + 2e^−^→2OH^−^. The bias of the Cr element is the reason for the selective corrosion of the alloy. As the value of *x* increases from 0.1 to 0.5, the number of Cr-rich FCC phases of the Al*_x_*Cr_1−*x*_CoFeNi HEAs decreases, and the cathode area decreases in parallel. Consequently, as the x-value of the Al elemental content increases, the self-corrosion current density decreases, the self-corrosion potential of the alloy gradually increases, the corrosion resistance is improved, and the Al_0.5_Cr_0.5_CoFeNi alloy exhibits the most favorable corrosion resistance.

## 4. Conclusions

The study aimed to elucidate the impact of aluminum (Al) on the microstructure and properties of CoCrFeNi high-entropy alloys (HEAs) in the Al*_x_*Cr_1−*x*_CoFeNi system (where *x* = 0.1, 0.2, 0.3, 0.4, 0.5). The mechanical alloying process was finalized by subjecting the amalgamated metal powder to ball milling at a rate of 350 rpm for a duration of 40 h. Subsequent to this, the AlCrCoFeNi powder was subjected to a plasma hot pressing process, employing a temperature of 1000 °C, a pressure of 31.2 MPa, and a holding time of 15 min. A comprehensive analysis of phase and microstructure, tensile properties, evaluation of hardness, friction and electrochemical testing leads to the following conclusions:

(1) The Al*_x_*Cr_1−*x*_CoFeNi HEAs are multiphase and comprise a Cr-rich FCC phase, a Cr-poor FCC phase, and a Cr-rich σ phase. The Al_0.1_Cr_0.9_CoFeNi alloy is similarly multiphase, with a Cr-rich FCC phase, a Cr-poor FCC phase, and a Cr-rich σ phase. As the aluminum content increases (*x* = 0.1~0.5), a portion of the FCC phase transforms into the BCC phase, while the weight percentage of the BCC phase increases from 0% to 32.9%. It can be observed that an imbalance of chromium aggregation occurs in the alloy, with this decreasing with increasing aluminum content.
(2) Tensile testing at room temperature indicates that the proportion of BCC phase in the alloy microstructure increases, resulting in enhanced tensile strength of the alloy and a reduction in its capacity for plastic deformation. Al_0.5_Cr_0.5_CoFeNi demonstrates the highest ultimate tensile strength (UTS) of 1163.14 MPa, while exhibiting the lowest elongation of 26.98%.
(3) The hardness of the alloys increased with the increase in the elemental content of Al. The Al_0.5_Cr_0.5_CoFeNi alloy exhibited the highest hardness, with a measured value of 412.6 HV. During the friction testing, the coefficient of friction of the alloys exhibited a gradual increase over time. This was accompanied by a transition in the wear mechanism, which initially manifested as adhesive wear, subsequently evolving into a combination of adhesive and abrasive wear, and finally, oxidative wear. The friction coefficient tends to stabilize when the oxide film stabilizes and reaches equilibrium with the wear process. The Al_0.1_Cr_0.9_CoFeNi alloy exhibits a minimum friction coefficient of 0.513 and a wear rate of 0.020 × 10^−3^ × mm^3^/Nm.
(4) Al*_x_*Cr_1−*x*_CoFeNi HEAs always contain both Cr-rich and Cr-poor phases. Therefore, in a 3.5 wt% NaCl solution, the alloy exhibits selective corrosion with the Cr-rich FCC phase acting as the cathode. An increase in aluminum content is associated with a decrease in the chromium-rich FCC phase content, a decline in the alloy’s self-corrosion current density, and an increase in its self-corrosion potential. The composition Al_0.5_Cr_0.5_CoFeNi has been shown to exhibit both the maximum *E*_corr_ and the minimum *I*_corr_, suggesting that it possesses optimal corrosion resistance.

## Figures and Tables

**Table 1:** Chemical composition of Al*_x_*Cr_1−*x*_CoFeNi HEAs (wt.%).

Alloy	Al	Co	Cr	Fe	Ni
Al*_x_*Cr_1−*x*_CoFeNi	1.21	26.40	20.94	25.06	26.40
Al*_x_*Cr_1−*x*_CoFeNi	2.44	26.70	18.82	25.34	26.70
Al*_x_*Cr_1−*x*_CoFeNi	3.71	27.00	16.66	25.63	27.00
Al*_x_*Cr_1−*x*_CoFeNi	5.00	27.31	14.44	25.93	27.31
Al*_x_*Cr_1−*x*_CoFeNi	6.32	27.63	12.18	26.23	27.63

**Table 2:** *VEC* of the elements, atomic size (Å), and Δ*H*_mix_ of the atomic pair (KJ/mol).

Elements	Co	Fe	Ni	Cr	Al
*VEC*	9	8	10	6	3
Atomic size	1.25	1.26	1.24	1.28	1.48
Co	-	−1	0	−4	−19
Fe		-	−2	−1	−11
Ni			-	−7	−22
Cr				-	−10
Al					-

**Table 3:** Calculated results of Al*_x_*Cr_1−*x*_CoFeNi characteristic parameters.

Alloy	∆*G*_mix_ (K·mol)	∆*H*_mix_ (KJ/mol)	*δ* (%)	*VEC*
Al_0.1_Cr_0.9_CoFeNi	1.47 R	−4.98	2.30	8.18
Al_0.2_Cr_0.8_CoFeNi	1.51 R	−6.15	3.05	8.06
Al_0.3_Cr_0.7_CoFeNi	1.54 R	−7.28	3.62	7.94
Al_0.4_Cr_0.6_CoFeNi	1.55 R	−8.35	4.08	7.72
Al_0.5_Cr_0.5_CoFeNi	1.56 R	−9.38	4.47	7.50

**Table 4:** EDS analysis of marked zones in Figure 5 (at%).

Alloy	Zone	Al	Cr	Fe	Co	Ni
Al_0.1_Cr_0.9_CoFeNi	1	3.25	17.86	23.54	26.78	28.57
Al_0.2_Cr_0.8_CoFeNi	2	8.82	22.43	18.01	25.50	25.23
	3	10.59	8.44	14.59	22.93	43.45
Al_0.3_Cr_0.7_CoFeNi	4	7.52	32.56	20.03	20.40	19.50
	5	20.83	12.02	24.54	22.97	19.64
Al_0.4_Cr_0.6_CoFeNi	6	11.55	23.27	12.23	32.09	20.87
	7	27.59	4.78	17.92	22.31	27.41
Al_0.5_Cr_0.5_CoFeNi	8	14.34	3.97	15.95	32.67	33.07
	9	47.12	6.51	13.86	17.25	15.26

**Table 5:** The mechanical properties of Al*_x_*Cr_1−*x*_CoFeNi HEAs.

Alloy	YS (MPa)	UTS (MPa)	Elongation (%)
Al_0.1_Cr_0.9_CoFeNi	403.93	702.01	43.01
Al_0.2_Cr_0.8_CoFeNi	437.35	737.70	39.21
Al_0.3_Cr_0.7_CoFeNi	634.69	863.71	36.57
Al_0.4_Cr_0.6_CoFeNi	578.45	933.90	32.03
Al_0.5_Cr_0.5_CoFeNi	664.22	1163.14	26.98

**Table 6:** Vickers hardness of Al*_x_*Cr_1−*x*_CoFeNi alloys.

Alloy	Hardness (HV)
Al_0.1_Cr_0.9_CoFeNi	311.66
Al_0.2_Cr_0.8_CoFeNi	324.60
Al_0.3_Cr_0.7_CoFeNi	360.34
Al_0.4_Cr_0.6_CoFeNi	403.42
Al_0.5_Cr_0.5_CoFeNi	412.60

**Table 7:** Abrasion rate *K* of Al*_x_*Cr_1−*x*_CoFeNi HEAs (10^−3^ × mm^3^/Nm).

Alloy	Al_0.1_Cr_0.9_CoFeNi	Al_0.2_Cr_0.8_CoFeNi	Al_0.3_Cr_0.7_CoFeNi	Al_0.4_Cr_0.6_CoFeNi	Al_0.5_Cr_0.5_CoFeNi
*K*	0.021	0.020	0.141	0.038	0.072

## Data Availability

The original contributions presented in this study are included in the article. Further inquiries can be directed to the corresponding author.
